# Individual-Level Prediction of Exposure Therapy Outcome Using Structural and Functional MRI Data in Spider Phobia: A Machine-Learning Study

**DOI:** 10.1155/2023/8594273

**Published:** 2023-08-22

**Authors:** Alice V. Chavanne, Charlotte Meinke, Till Langhammer, Kati Roesmann, Joscha Boehnlein, Bettina Gathmann, Martin J. Herrmann, Markus Junghoefer, Luisa Klahn, Hanna Schwarzmeier, Fabian R. Seeger, Niklas Siminski, Thomas Straube, Udo Dannlowski, Ulrike Lueken, Elisabeth J. Leehr, Kevin Hilbert

**Affiliations:** ^1^Department of Psychology, Humboldt-Universität zu Berlin, Berlin, Germany; ^2^Université Paris-Saclay, INSERM U1299 “Trajectoires développementales et psychiatrie”, CNRS UMR 9010 Centre Borelli, Ecole Normale Supérieure Paris-Saclay, France; ^3^Institute of Clinical Psychology and Psychotherapy, University of Siegen, Germany; ^4^Institute for Biomagnetism and Biosignalanalysis, University of Münster, Germany; ^5^Institute of Psychology, Unit of Clinical Psychology and Psychotherapy in Childhood and Adolescence, University of Osnabrück, Germany; ^6^Institute for Translational Psychiatry, University of Münster, Germany; ^7^Institute of Medical Psychology and Systems Neuroscience, University of Münster, Germany; ^8^Department of Psychiatry, Psychosomatics, and Psychotherapy, Center for Mental Health, University Hospital of Würzburg, Germany; ^9^Otto-Creutzfeld Center for Cognitive and Behavioral Neuroscience, University of Münster, Germany; ^10^Department of Psychiatry and Neurochemistry, Institute of Neuroscience and Physiology, University of Gothenburg, Sweden

## Abstract

Machine-learning prediction studies have shown potential to inform treatment stratification, but recent efforts to predict psychotherapy outcomes with clinical routine data have only resulted in moderate prediction accuracies. Neuroimaging data showed promise to predict treatment outcome, but previous prediction attempts have been exploratory and reported small clinical sample sizes. Herein, we aimed to examine the incremental predictive value of neuroimaging data in contrast to clinical and demographic data alone (for which results were previously published), using a two-level multimodal ensemble machine-learning strategy. We used pretreatment structural and task-based fMRI data to predict virtual reality exposure therapy outcome in a bicentric sample of *N* = 190 patients with spider phobia. First, eight 1st-level random forest classifications were conducted using separate data modalities (clinical questionnaire scores and sociodemographic data, cortical thickness and gray matter volumes, functional activation, connectivity, connectivity-derived graph metrics, and BOLD signal variance). Then, the resulting predictions were used to train a 2nd-level classifier that produced a final prediction. No 1st-level or 2nd-level classifier performed above chance level except BOLD signal variance, which showed potential as a contributor to higher-level prediction from multiple regions across the brain (1st-level balanced accuracy = 0.63). Overall, neuroimaging data did not provide any incremental accuracy for treatment outcome prediction in patients with spider phobia with respect to clinical and sociodemographic data alone. Thus, we advise caution in the interpretation of prediction performances from small-scale, single-site patient samples. Larger multimodal datasets are needed to further investigate individual-level neuroimaging predictors of therapy response in anxiety disorders.

## 1. Introduction

Anxiety disorders are amongst the most prevalent mental disorders [1] with a considerable burden of disease [2]. Current response rates to cognitive-behavioural therapy (CBT) as a first-line treatment average at 50% or lower for most anxiety disorders [3, 4]. Prospectively distinguishing treatment-responding from nonresponding patients could help guide clinical decisions and improve prognosis [5]. Machine-learning approaches can predict at the individual level on unseen samples and are well-suited for predicting individual therapeutic outcomes, particularly with the high-dimensional data collected in clinical research and practice [6]. A promising literature on machine-learning outcome prediction has emerged across mental disorders (see [7] for a general review), including a rapidly increasing number of psychotherapy outcome prediction studies [8].

However, recent large-scale efforts to predict individual-level psychotherapy treatment outcomes for patients with anxiety disorders based on routine clinical data alone resulted only in moderate prediction accuracies [9–11]. Neuroimaging data has shown promise to predict treatment outcomes for patients with anxiety disorders in previous attempts, but those have been exploratory and reported small clinical sample sizes [7].

To our knowledge, two studies conducted individual CBT outcome prediction using task-based fMRI in patients with panic disorder [12, 13], four in patients with social anxiety disorder [14–16], and one in a mixed sample of patients with panic disorder or generalized anxiety disorder [17] (see [8] for a recent review). However, no study had a sample with *N* > 60. It has been reported that studies using small sample sizes present a considerable risk of overestimating prediction performance in part because they are limited to much less robust cross-validation schemes [18–20]. A recent review encouraged the use of larger sample sizes to disentangle the contribution of neuroimaging data to psychotherapy response prediction from the effect of small sample sizes on reported prediction performance [8].

In all but one of the above CBT outcome prediction studies, predictive features were extracted from symptom-related fMRI tasks. Indeed, symptom-related task-based functional activation and connectivity are commonly used in anxiety disorder studies, and they, along with structural MRI, have been associated with the prospective treatment response of patients with anxiety disorders at the group level (see [21] for a review). Graph-theoretical measures derived from functional connectivity, reported to have overall good reproducibility [22], have also been used in recent years for fine-tuned investigation of functional network dysfunctions in anxiety disorders [23, 24]. Additionally, BOLD signal variability measures have recently been reported as promising individual-level predictors for therapeutic outcomes in anxiety disorders [14, 16].

Therefore, the aim of the present study was to build upon previous literature using a fairly large, bicentric, and clinically well-characterized sample of patients with spider phobia to investigate the incremental performance of structural MRI and symptom-related task-based fMRI measures over routine clinical data in predicting psychotherapy outcome with a state-of-the-art ensemble machine-learning pipeline. We hypothesized that structural and task-based (f)MRI measures would predict posttreatment and 6-month follow-up psychotherapy outcomes significantly beyond the chance level and that an ensemble approach using clinical, sociodemographic, and neuroimaging modalities would produce higher predictive performance than clinical and sociodemographic data alone.

## 2. Methods

### 2.1. Dataset and Sample Description

The bicentric clinical study SPIDER-VR was part of the Transregional Collaborative Research Centre 58 “Fear, Anxiety, Anxiety Disorders” (clinical trial registration at clinicaltrials.gov: NCT03208400). It includes a sample of untreated patients with spider phobia according to DSM-IV criteria [25] aged 18-65 without major comorbidities (low to moderate depression was tolerated unless currently treated, as well as other animal phobias) and with a total Spider Phobia Questionnaire (SPQ) [26] score > 19 (clinical cut-off). See [27] for a complete study description and [10, 28–31] for other studies using the SPIDER-VR data. Bicentric recruitment was conducted in Würzburg (WÜ) and Münster (MS), Germany. The SPIDER-VR study protocol has been reviewed by the Ethics Committees of the Medical Faculties of Münster University (proposal 216-212-b-S) and Würzburg University (proposal 330/15), and written informed consent was obtained from all participants.

Clinical and neuroimaging data were acquired before treatment. Patients were then invited for a one-session virtual reality exposure therapy (VRET), and the primary outcome (responder/nonresponder status at posttreatment and at 6-month follow-up [FU]) was based on a 30% SPQ score reduction between pretreatment and posttreatment or FU, respectively.

Of note, clinical effects of VRET and prediction results based only on sociodemographic and clinical data using the original sample of SPIDER-VR patients (*N* = 171 for the prediction) have been previously published [10]. Herein, we investigate the incremental value of neuroimaging data using an extended sample of SPIDER-VR patients (due to the continuation of patient recruitment in MS). In this extended sample, *N* = 211 patients had complete posttreatment data, but twelve did not have available functional MRI data, three were excluded for structural MRI artefacts or abnormalities, five were excluded due to substantial movement during the task, and one was excluded due to absent visual activation (see the feature extraction section below for quality control details). Thus, *N* = 190 patients in total (81.6% overlap with the sample in [10]) were included for analysis at posttreatment (see Table 1 for sample description). Primary treatment response (30% SPQ score reduction between pretreatment and posttreatment) was observed in 54% of patients. A sample description of follow-up responders and nonresponders is presented in Supplementary Table 1, and prediction analyses on the primary outcome at follow-up are presented in Supplementary results.

### 2.2. MRI Data Acquisition

The full acquisition procedure is described in [27]. Briefly, all scans were obtained with 3 T MRI scanners (WÜ: Siemens Skyra, MS: Siemens Prisma). A structural T1 dataset was collected using an MPRAGE acquisition sequence (256 × 256 × 176 matrix, FOV = 256, voxel size = 1 × 1 × 1 mm, TE = 2.26 ms [WÜ], TE = 2.28 ms [MS], TR = 1.9 s [WÜ], TR = 2.13 s [MS], flip angle = 9° [WÜ], flip angle = 8° [MS]). Functional images were collected with a T2⁣^∗^ weighted EPI sequence in ascending order (64 × 64 × 33 matrix, FOV = 210, voxel size = 3.3 × 3.3 × 3.8 mm, slice thickness = 3.8 mm, 10% slice gap, TE = 30 ms [WÜ], TE = 29 ms [MS], TR = 2.0 s, flip angle = 90°). Slices covered the whole brain and were positioned transaxially parallel to the anterior–posterior commissural line with a tilted angle of 20°. Stimuli were presented via MR-compatible LCD goggles (WÜ) or via a back-projection monitor (MS).

### 2.3. Sustained and Phasic Fear (SPF) Task

The SPF task is a suitable paradigm to measure activation in relevant networks for fear processing and has been used in previous literature to detect significant differences in functional activation in patients with spider phobia compared to nonanxious controls during both phasic fear and sustained fear conditions [32]. Of note, analyses of the activation patterns during sustained and phasic fear in patients with spider phobia revealed increased anterior cingulate cortex activation during sustained rather than phasic fear, whereas amygdala and insula activation were of particular relevance for phasic fear processing (see Breede et al., in prep.). Though lacking a healthy control group, these results can be seen in line with the results in [32].

The task employed a block design including 15 active and 14 inactive blocks. During inactive blocks, a fixed dot was displayed in the middle of the screen for 15 s. Active blocks included 10 successive trials in which a picture was shown for 1.7 s and followed by 300 ms of fixation dot. Each active block was followed by an inactive block. Active blocks were split between three fear conditions in pseudorandomized order: (1) a sustained fear condition, during which participants were informed that a spider could appear; pictures of empty rooms were shown, and in three of the five sustained fear blocks, a spider was shown instead of an empty room in the last quarter of the block; (2) a phasic fear condition in which participants were told they would see spiders and were shown spider pictures; and (3) a no fear (safety) condition, during which participants were shown pictures of empty rooms. After each active block, participants had to rate their experience from very pleasant to very unpleasant. The total task duration was 9:45 min.

### 2.4. Feature Extraction

Sociodemographic, clinical, and structural MRI and fMRI data were used as features, and each is described in detail below. The fMRI data provided phasic and sustained fear activation, BOLD variance, and functional connectivity features, and graph-theoretical features were then derived from functional connectivity matrices.

#### 2.4.1. Sociodemographic Data and Clinical Questionnaires

The pretreatment sociodemographic and clinical features included in the prediction analysis were previously described in [10]. Briefly, they included age; gender; years of education; age at phobia onset; family history of mental health conditions; comorbidities; lifetime suicidal intent; smoking; consumption of alcohol, cannabis, and coffee; distance; and salience of a standardized behavioural avoidance test (a live bird spider was placed in a closed box that patients had to drag as close as possible to themselves), as well as sum scores and subscales of a battery of anxiety-relevant questionnaires such as the SPQ, State-Trait Anxiety Inventory [33], questionnaire regarding the fear of spiders [34], and a questionnaire regarding the disgust and fear of spiders [35] (see Supplementary methods for a complete list of the questionnaires used).

#### 2.4.2. Structural MRI Data

Structural data processing and quality control were conducted with Freesurfer [36] in accordance with ENIGMA protocols (https://enigma.ini.usc.edu/protocols/imaging-protocols/). Total intracranial volume was extracted, cortical surface area and cortical thickness were extracted for 68 cortical ROIs of the Desikan-Killiany atlas [37], and volume was extracted for 16 subcortical ROIs from the Freesurfer automatic segmentation [36]. A visual control was conducted for segmentation failure or substantial over- or underestimation, and the data from the affected regions were excluded.

#### 2.4.3. Task-Based fMRI Data

The data preprocessing and processing steps described below were conducted for each subject individually. Initial visual quality control was conducted for structural and functional data, and subjects with excessive noise, motion artefacts, or abnormal brain anatomy were excluded (as mentioned above, *N* = 3 were excluded for structural MRI artefacts or abnormalities, and *N* = 5 were excluded due to substantial movement during the task).

Task fMRI data were preprocessed with SPM12 and the associated toolbox CONN [38]. Functional volumes were realigned and unwarped, and potential outlier scans were detected in CONN with conservative parameters (i.e., flagging scans with within-subject global BOLD signal change ≥ 3 standard deviations or framewise displacement of 0.5 mm). Volumes were then segmented and normalized onto MNI template space, then smoothed with 8 mm full width at the half-maximum Gaussian kernel. Subjects with movement-correction realignment parameters ≥ 3.3 mm (initial voxel size) in any direction were excluded. The absence of occipital visual activation in the active vs. inactive block contrast was also an exclusion criterion. Functional measures described below were extracted for a set of 30 bilateral anxiety-relevant ROIs derived from recent meta-analyses and reviews [21, 39] to restrict the dimensionality of features for the main prediction analysis (see Supplementary methods for the complete list of ROIs).

Given that some of the previous literature reported widespread brain regions to have predictive value in psychotherapy outcome prediction [12], an exploratory prediction analysis was also conducted in which functional measures were extracted for every ROI in the CONN default atlas, covering the whole brain (combining cortical and subcortical areas from the FSL Harvard-Oxford atlas and the AAL cerebellar areas for a total of 132 ROIs).

*(1) Activation Features*. Condition effects were modeled using the general linear model in SPM with separate conditions for phasic fear, sustained fear, no fear, instructions, rating, and inactive blocks separately to map the entire experimental space. The six movement-correction parameters from the realignment procedure were used as regressors of no interest. Default 1st-level SPM analysis parameters were used. Three contrasts of interest were computed per subject (phasic fear vs. no fear, sustained fear vs. no fear, and active blocks vs. inactive blocks). For each contrast per subject, the Marsbar toolbox [40] was then used to extract median effect sizes in every ROI, which were included as features.

*(2) BOLD Variance Features*. All the preprocessing described above was kept identical apart from smoothing, which was absent. According to [41], realigned unwarped normalized unsmoothed volumes, as well as the 1st-level SPM model described above, were used as input in VarTbx (https://github.com/LNDG/vartbx), and a boxcar model was used to model the task design. To correct block offsets from the concatenated blocks, all blocks were normalized to have a four-dimensional mean of 100. The block mean was then substracted from each voxel, and the detrended variance of each condition was extracted voxelwise, producing whole-brain BOLD variance maps. For the phasic fear, sustained fear, and no fear conditions, the Marsbar toolbox was then used to extract the average variance in every ROI, which were included as features.

*(3) Functional Connectivity Features*. Preprocessed functional volumes were denoised with the standard CONN pipeline (linear regression of potential confounding effects including noise components from cerebrospinal fluid and white matter and temporal band-pass filtering [0.008-0.09 Hz]). ROI-to-ROI task-modulated effective connectivity matrices were computed with generalized psychophysiological interaction (gPPI) for all ROIS both in the phasic fear and sustained fear conditions and were included as features.

*(4) Graph-Theoretic Connectivity Features*. The abovementioned gPPI matrices were used in the BCT toolbox [42]. The gPPI matrices for phasic and sustained fear (i.e., weighted directed graphs) were thresholded with *r* = 0.3 to avoid spurious edges. All global and ROI-specific metrics available in the toolbox for directed graphs were extracted (degree, strength, density, clustering coefficient, transitivity, global and local efficiency, assortativity, characteristic path length, betweenness centrality, *K*-coreness centrality, flow coefficient, as well as the fingerprint, intensity, and coherence of structural and functional motifs), with the exception of community-related metrics (due to the varying number of communities detected among subjects preventing their use as comparable predictive features), and included as features.

### 2.5. Ensemble Machine-Learning Prediction

**Figure 1:**
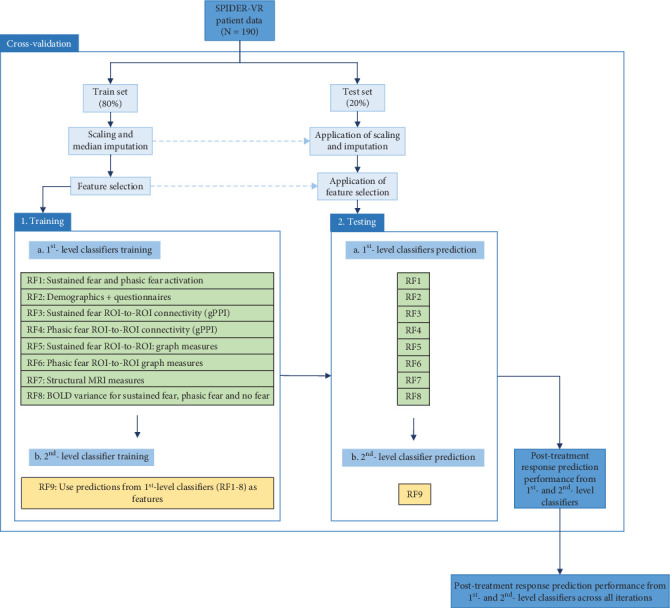
Ensemble machine-learning classification pipeline. RF: random forest; ROI: region of interest; gPPI: generalized psychophysiological interaction; MRI: magnetic resonance imaging; BOLD: blood-oxygen-level-dependent.

All prediction analyses were conducted with scikit-learn (version 1.1.1) in Python. A binary classification prediction between responders (*N* = 103) and nonresponders (*N* = 87) at posttreatment was conducted using an ensemble learning approach, with eight 1st-level classifiers each using different feature modalities (demographic and clinical questionnaires, functional activation, gPPI connectivity for both phasic and sustained fear, gPPI-derived graph-theoretic metrics, and BOLD variance; see Figure 1) from which the output (i.e., predictions) was used as a feature by a 2nd-level classifier, the latter producing the final prediction. Random shuffle cross-validation was repeated 100 times with an 80-20 train-test split used in every iteration and included scaling, median imputation, and feature selection using a logistic regression stochastic gradient descent learning classifier with mean feature importance as selection threshold (log-loss, elastic net penalisation, grid search tuning of l1 ratio between 0 and 1 with default 5-fold nested cross-validation; all other default classifier parameters were kept identical). All classifiers used were random forests (1000 estimators, out-of-bag score true, class weight balanced, all other default parameters kept identical). An alternative 2nd-level classifier (soft voting with the sum of 1st-level predictions) was also examined for completeness.

The mean performance metrics across the 100 cross-validation folds are reported in the results section. A corrected resampled *t*-test [43, 44] between the balanced accuracy of classifiers of interest and the one of a dummy classifier always predicting the majority class was used to investigate above-chance classification accuracies.

To explore individual feature contribution to predictions, the Shapley additive explanation (SHAP) module was used [45]. SHAP uses a game-theoretic approach to assign an importance value to each feature for an individual prediction.

## 3. Results

### 3.1. 1st-Level Posttreatment Outcome Prediction Results

**Figure 2:**
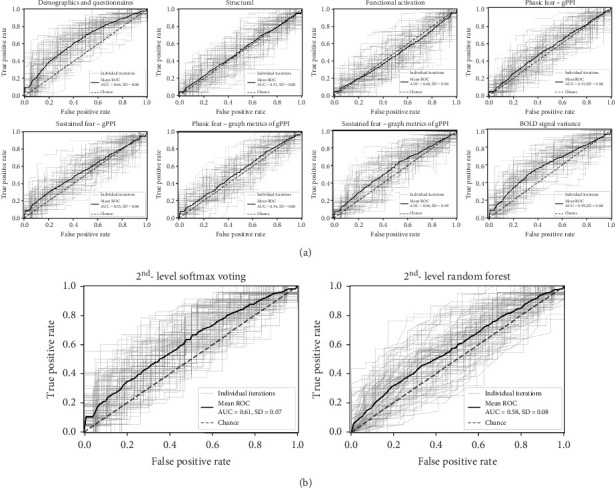
Area under the receiving operating curves for treatment outcome classification. (a) 1st-level classification results. (b) 2nd-level classification results. gPPI: generalized psychophysiological interaction.

The main prediction analysis of posttreatment outcome based on demographic and clinical questionnaires resulted in a balanced accuracy = 0.60 (SD = 0.07) and AUROC = 0.64 (SD = 0.08) (Table 2, Figure 2(a)). No significant difference emerged between the sociodemographic and clinical classifier and the dummy classifier using the corrected resampled *t*-test.

The main prediction analysis based on structural MRI measures, functional activation, gPPI connectivity, gPPI-derived graph metrics for both phasic and sustained fear conditions, and BOLD signal variance did not perform above chance level (balanced accuracy ranging from 0.48 to 0.55, AUROC ranging from 0.48 to 0.59).

The exploratory prediction analysis, in which functional features were derived from ROIs across the whole brain instead of an a priori selected set, produced similar results except for the 1st-level BOLD signal variance classifier, which resulted in a balanced accuracy = 0.63 (SD = 0.07) and AUROC = 0.67 (SD = 0.08) (Table 2, Supplementary Figure 1A). A significant performance difference was found between the BOLD variance classifier and the dummy classifier using the corrected resampled *t*-test (*p* = 0.041).

**Figure 3:**
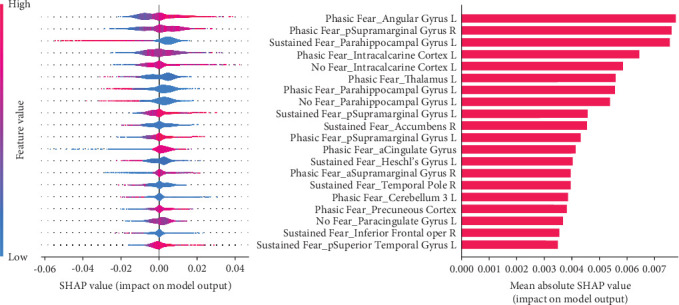
The Shapley (SHAP) values and feature importance of the 1st-level variance classifier in the responder (*N* = 103) vs. nonresponder (*N* = 87) prediction using functional features across ROIs covering the whole brain. Positive SHAP values indicate a contribution of feature value in favour of the positive class prediction (future responder); negative Shapley values are in favour of the negative class prediction (future nonresponder). Larger absolute Shapley values indicate a larger impact on the model output. The 20 most contributing features are shown and ranked in decreasing order of mean absolute SHAP value. Horizontal violin plots on the left represent the distribution of all individuals in the test set across all cross-validation iterations. For each feature, relative values are represented on the left by a color gradient.

Features that contributed most to this variance classifier prediction varied across conditions and brain regions, including, for instance, BOLD variance in the right supramarginal gyrus, left parahippocampal and angular gyri, and left intracalcarine cortex (Figure 3).

### 3.2. 2nd-Level Posttreatment Outcome Ensemble Prediction Results

The 2nd-level classifiers using the prediction probabilities of all 1st-level classifiers as input features failed to predict treatment outcome above the chance level in the main prediction analysis (Figure 2(b)). The 2nd-level voting classifier prediction resulted in a balanced accuracy = 0.55 (SD = 0.06) and AUROC = 0.61 (SD = 0.07). The 2nd-level random forest classifier resulted in a balanced accuracy = 0.54 (SD = 0.07) and AUROC = 0.58 (SD = 0.08).

Comparable results were obtained with the exploratory analysis in which functional features were derived from ROIS across the whole brain instead of an a priori selected set, with the voting classifier resulting in a balanced accuracy = 0.54 (SD = 0.06) and AUROC = 0.62 (SD = 0.08) and the random forest classifier resulting in a balanced accuracy = 0.52 (SD = 0.06) and AUROC = 0.58 (SD = 0.08) (Supplementary Figure 1B).

## 4. Discussion

The present study investigated the incremental predictive value of neuroimaging data with respect to clinical data alone for individual-level psychotherapy outcome prediction in patients with spider phobia. Contrary to expectations, prediction performance did not go beyond chance level for all distinct data modalities except BOLD variance, and the contribution of (f)MRI measures to the prediction did not outperform clinical and sociodemographic data alone. At posttreatment, clinical questionnaires and BOLD signal variance derived from ROIs across the whole brain showed the potential to contribute to higher-level ensemble prediction with a balanced accuracy of 0.60 and 0.63, respectively. No predictive contribution was found for any data modality at follow-up (see Supplementary results).

### 4.1. Perspective on Prediction Performance

Overall, our findings challenge the existing literature reporting above-chance predictive accuracies for machine-learning psychotherapy outcome prediction using neuroimaging data in patients with anxiety disorders [12–17, 46]. However, they echo a more recent body of methodological work underlining that, despite initial promise in the field, prediction accuracies for patient classification based on medical imaging features appear to be decreasing as sample sizes increase, perhaps reflecting unwitting biases in performance evaluation, overhyping, and cross-validation error bars in the neuroimaging literature [19, 47–49]. The importance of general sample size and the size of test sets in particular to guard against misestimation of prediction accuracy was underlined in a study using a very large sample of patients with depression to mimic small-scale sampling results in machine-learning classification using structural neuroimaging [18]. In line with this, another recent prospective prediction of pharmacotherapy outcome in a relatively large sample of patients with depression using baseline cross-sectional functional MRI connectivity yielded no prediction above chance level, although using changes in connectivity from baseline to week two as predictive features instead yielded accuracies up to 0.696 [50]. This study pointed out that many previous studies reporting high classification or prediction accuracies were based on single-site, small samples of patients that do not generalize well, and that although more heterogeneous and larger multisite datasets may yield lower prediction performances, they were more representative of the target population. While our sample was bicentric and fairly larger than previously published studies, it is not large per machine-learning standards. Efforts to collaboratively build multisite samples with very large sizes, such as the ENIGMA consortium initiative [51], should be bolstered to address this recurring concern. The performance of machine-learning models can also vary depending on the initial choice of various prediction pipeline elements and can also be affected by the incorporation of distinct data modalities in the prediction.

The clinical demographic classifier showed close performance to a previous prediction study using the original SPIDER-VR sample [10]; however, it was not significantly above the chance level in our study (possible causes include distinct balancing and cross-validation strategies between the two studies and the use of an extended sample herein). The BOLD variance classifier did reach above-chance predictive performance on its own at posttreatment in our exploratory analysis with features extracted from ROIs across the whole brain but with moderate performance. It could be a promising contributor to 2nd-level prediction alongside other feature modalities. Indeed, in the field of neuroimaging, interest in BOLD signal variability has been increasing with mounting evidence that it could be a promising correlate of cognitive performance with good measurement reliability and a more flexible brain state allowing more accurate processing, complementary to the traditional BOLD signal mean [52–54]. BOLD signal variability has also been reported to differ significantly between patients with generalized anxiety disorder and healthy controls in widespread brain regions, with a nonlinear relationship between anxiety level and variability, showing promise as a potential clinical biomarker [55]. Critically, given the recent and sparse literature on the predictive value of BOLD variability both in resting-state and task-based fMRI in anxiety disorders [14, 16], our study supports further investigation of BOLD variability as a predictive feature of clinical outcome.

Additionally, other psychological, neuroimaging, and biological measures could also be explored for individual-level predictive purposes. For instance, early response to psychotherapy is a well-established group-level predictor of posttreatment response in patients with internalizing disorders [56–60], and early functional connectivity variation during psychotherapy was also reported to be predictive of individual-level clinical outcome [50]. The promise of ecological momentary assessments to measure symptom dynamics and inform clinical decisions in patients with anxiety disorders has been recently underlined [61]. Epigenetic markers have also been noticed as promising group-level prospective biomarkers for psychotherapy response in patients with stress-related and anxiety disorders in the recent literature [62].

Based on our results as well as the previously mentioned literature, we encourage caution in the interpretation of promising neuroimaging prediction results with small patient sample sizes. Further investigation with large and multisite samples is still needed to elucidate the potential contribution of (f)MRI measures to the prediction of anxiety disorder therapy response.

### 4.2. Strengths

The clinical outcome prediction was based on targeted, anxiety-specific standardized questionnaires and on multimodal MRI data, including both structural and several functional neuroimaging measures. Additionally, our investigation included a state-of-the-art machine-learning pipeline designed to incorporate the respective predictive contributions of distinct data modalities and to maximize feature interpretability.

### 4.3. Limitations

Limitations to our study include the ROI-based approach, which induced a loss of information in comparison to more fine-tuned, voxel-wise approaches, particularly for variability-based measures. However, this approach was necessary to keep a more reasonable feature dimensionality and reduce overfitting.

Our sample also included quite homogeneous spider-phobic patients without major comorbidities and might not be fully representative of a diverse clinical population of patients with anxiety disorders. It was, however, crucial for internal validity to investigate the clinical effects of VRET in previous SPIDER-VR publications.

## 5. Conclusion

The present study found no evidence of an incremental contribution of structural MRI and symptom-relevant task-based fMRI measures to psychotherapy outcome prediction in a fairly large and bicentric sample of patients with spider phobia, with the exception of BOLD signal variance which performed moderately above chance. As such, our findings invite further investigation of BOLD signal variability as a contributor to higher-level prediction. However, even the BOLD signal variability prediction performance was lower than in previous single-site, small-sample literature. Thus, the present study also warrants caution in interpreting previous small-scale psychotherapy outcome prediction studies and underlines the need for larger, multisite, and multimodal datasets to further examine the predictive contribution of neuroimaging measures to psychotherapy response in anxiety disorders.

## Figures and Tables

**Table 1:** Pretreatment sample description of posttreatment responders and nonresponders.

Variables	Posttreatment responders	Posttreatment non-responders	*p* value
	*N* = 103	*N* = 87	
Demographic characteristics at pretreatment			
Gender (m/f)	13/90	12/75	n.s.
Site distribution	WÜ:54	WÜ:29	1.3*e*-2⁣^∗^
	MS:49	MS: 58	
Age (SD)	26.6 (7.5)	30.4 (9.9)	3.6*e*-3⁣^∗^
Years of education (SD)	14.6 (3.0)	14.5 (3.0)	n.s.
Clinical characteristics at pretreatment			
Age of onset spider phobia (SD)	7.2 (4.6)	6.4 (4.6)	n.s.
Comorbid depression, *n* (%)	3 (2.9)	3 (3.4)	n.s.
SPQ (SD)	20.8 (3.5)	19.9 (4.2)	n.s.
LSAS (SD)	22.4 (16.0)	26.1 (18.6)	n.s.
ASI-3 (SD)	14.6 (7.0)	16.2 (8.1)	n.s.
STAI trait (SD)	34.7 (8.4)	35.8 (8.2)	n.s.
BDI-II total (SD)	3.0 (3.6)	3.3 (3.9)	n.s.
UI-18 (SD)	37.5 (12.4)	39.9 (13.3)	n.s.
Promis-specific phobia (SD)	11.3 (8.4)	11.0 (8.9)	n.s.
FEAS anxiety (SD)	102.3 (13.5)	100.6 (10.8)	n.s.
FAS (SD)	83.6 (12.7)	83.3 (11.7)	n.s.
Final BAT distance (cm) (SD)	175.7 (61.4)	158.1 (69.2)	n.s.
Posttreatment			
SPQ (SD)	13.2 (2.4)	17.8 (2.0)	<2.2*e*-16⁣^∗^
Follow-up			
SPQ (SD)	12.2 (2.8)	15.3 (3.2)	1.5*e*-10⁣^∗^

Statistical tests were two-sided *t*-test for continuous variables and chi-squared tests for categorical variables. WÜ: Würzburg; MS: Münster; SPQ: spider fear questionnaire; LSAS: Liebowitz Social Anxiety Scale; ASI-3: Anxiety Sensitivity Scale 3; STAI: State-Trait Anxiety Inventory; BDI-II: Beck Depression Inventory II; UI-18: Unsicherheitsintoleranz (intolerance of uncertainty) 18 scale; PROMIS: Patient-Reported Outcomes Measurement Information System (PROMISPHO: specific phobia); FEAS: Fragebogen zur Ekel und Angst vor Spinnen (questionnaire regarding disgust and fear of spiders); FAS: Fragebogen zur Angst von Spinnen (questionnaire regarding the fear of spiders); BAT: behavioural avoidance test.

**Table 2:** Prediction results of the 1st-level classifiers of posttreatment response (*N* = 103 responders vs. *N* = 87 nonresponders).

1st-level classifier	Balanced accuracy (SD)	AUROC (SD)
Functional features extracted from a priori selected 30 ROIs		
Demographic and questionnaire data	0.60 (0.07)	0.64 (0.08)
Structural MRI	0.51 (0.07)	0.51 (0.08)
Functional activation	0.48 (0.08)	0.48 (0.09)
Phasic fear gPPI	0.52 (0.06)	0.53 (0.08)
Sustained fear gPPI	0.51 (0.06)	0.55 (0.08)
Phasic fear graph measures	0.52 (0.07)	0.54 (0.08)
Sustained fear graph measures	0.54 (0.07)	0.56 (0.09)
BOLD variance	0.55 (0.07)	0.59 (0.08)
Functional features extracted from ROIs across the whole brain (exploratory)		
Demographic and questionnaire data	0.60 (0.07)	0.64 (0.08)
Structural MRI	0.51 (0.07)	0.51 (0.08)
Functional activation	0.51 (0.06)	0.52 (0.08)
Phasic fear gPPI	0.49 (0.05)	0.49 (0.10)
Sustained fear gPPI	0.49 (0.05)	0.49 (0.09)
Phasic fear graph measures	0.48 (0.06)	0.50 (0.07)
Sustained fear graph measures	0.47 (0.06)	0.49 (0.08)
BOLD variance	0.63⁣^∗^ (0.07)	0.67 (0.08)

ROI: region of interest; AUROC: area under the receiving operator curve; gPPI: generalized psychophysiological interaction. ⁣^∗^*p* < 0.05, corrected resampled *t*-test against the accuracy of a dummy classifier, two-tailed.

## Data Availability

The prediction analysis code, as well as the code for neuroimaging measures extraction, has been made freely available online (https://github.com/avchavanne/SpiderPhobia_Treatment_response_prediction_multimodal).

## Supplementary Materials

Supplementary Materials Main file includes (1) a Supplementary Methods section providing a complete list of questionnaires used as features in the prediction, a complete list of the a priori regions of interest used to extract neurofunctional measures, and describing alternative prediction strategies used; (2) a Supplementary Results section providing performance metrics of the prediction of psychotherapy response after a 6-month follow-up and those obtained using alternative prediction strategies; (3) Supplementary Table 1 presenting pretreatment patient sample description of follow-up responders and nonresponders; (4) Supplementary Figure 1 presenting the area under the receiving operating curves for treatment outcome classification using functional features extracted for regions of interest across the whole brain; and (5) a Supplementary References section supporting the Supplementary Methods section.
