# Supplementary material for: Individual-Level Prediction of Exposure Therapy Outcome Using Structural and Functional MRI Data in Spider Phobia: A Machine-Learning Study
Source: Depress Anxiety. 2023 Aug 22;2023:8594273. doi: 10.1155/2023/8594273 (PMC11921848; doi:10.1155/2023/8594273)
Supplement: Supplementary Materials — Main file includes (1) a Supplementary Methods section providing a complete list of questionnaires used as features in the prediction, a complete list of the a priori regions of interest used to extract neurofunctional measures, and describing alternative prediction strategies used; (2) a Supplementary Results section providing performance metrics of the prediction of psychotherapy response after a 6-month follow-up and those obtained using alternative prediction strategies; (3) Supplementary Table 1 presenting pretreatment patient sample description of follow-up responders and nonresponders; (4) Supplementary Figure 1 presenting the area under the receiving operating curves for treatment outcome classification using functional features extracted for regions of interest across the whole brain; and (5) a Supplementary References section supporting the Supplementary Methods section. [file 8594273.f1.docx]

**Supplementary Methods**

Clinical questionnaires

The complete list of questionnaires used for the clinical and sociodemographic classifier included: Specific Phobia Questionnaire (1), Anxiety Sensitivity Index (2), Liebowitz Social Anxiety Scale (3), Uncertainty Intolerance questionnaire (4), State-Trait Anxiety Inventory (5), Questionnaire regarding the fear of spiders (Fragebogen zur Angst vor Spinnen) (6), Questionnaire regarding the disgust and fear of spiders (Fragebogen zu Ekel und Angst vor Spinnen) (7), Positive And Negative Affect Schedule (8), Penn State Worry Questionnaire (9), Social Phobia and Anxiety Inventory (10), Agoraphobic Cognitions Questionnaire (11), Beck Depression Inventory (12), General scale of depression (Allgemeine Depressionsskala) (13), Short questionnaire about stresses and strains (Kurzer Fragebogen zur Belastungen) (14), Patient-Reported Outcome Information System (15), Beck Anxiety Inventory (16), Brief COPE (17), Childhood Trauma Questionnaire (18), General Self-Efficacy scale (19).

A-priori selected set of ROIs

ROIs included the amygdala, hippocampus, anterior and posterior insula, periaqueductal gray, bed nucleus of the stria terminalis, the dorsal, pregenual and subgenual anterior cingulate cortex, as well as the dorsomedial, ventromedial, dorsolateral and orbitofrontal cortices (all were taken from the Brainnetome atlas (20) with the exception of periaqueductal gray, taken from Keuken et al. (21), and bed nucleus of the stria terminalis, taken from Neudorfer et al. (22)).

Follow-up outcome ensemble prediction

Sustained treatment outcome (30% SPQ score reduction between pre-treatment and FU) after 6 months was also analysed, including N = 183 patients with a response rate of 78%. Additional prediction analyses were conducted with an identical classification pipeline and features to the main analysis, to investigate the prospective prediction of responders (N = 143) vs. non-responders (N = 40) at FU.

Alternative prediction strategies

With all other parameters kept identical, prediction analyses were re-performed *a posteriori* using a decision tree classifier as feature selection (with scikit-learn default parameters) instead of the gradient descent learning classifier.

The prediction analyses were also re-performed using alternative structural data, which was re-extracted from all subjects using the CAT12 toolbox (https://neuro-jena.github.io/cat//). Regional cortical thickness and gyrification were extracted from the Destrieux atlas, alongside regional volumes from the neuromorphometrics atlas.

Supplementary Results

Follow-up outcome ensemble prediction

The main prediction analysis of follow-up treatment primary outcome based on any modality (functional activation, demographic and clinical data, functional connectivity gPPI and gPPI-derived graph metrics, structural measures, BOLD variance) did not perform above chance level (balanced accuracy = 0.5 for all 1^st^-level classifiers, AUROC ranging between 0.47 and 0.56). Similarly, both soft voting and Random Forest 2^nd^-level classifiers did not predict follow-up treatment outcome above chance level (balanced accuracy = 0.5 (SD = 0) for both, AUROC = 0.49 (SD = 0.11) and 0.5 (SD = 0.02) respectively).

The exploratory prediction analysis resulted in similar results, with no performance above chance level from any 1^st^- or 2^nd^-level classifier.

Additional machine-learning predictions

Re-performing the analyses using a non-linear (decision tree) classifier to select features did not impact prediction performance (AUROC of 1^st^-level classifiers ranging between 0.48 and 0.64; balanced accuracy between 0.49 and 0.60; 2^nd^-level voting classifier AUROC = 0.59 (SD = 0.09), balanced accuracy = 0.55 (SD = 0.07); 2^nd^-level Random Forest classifier AUROC = 0.58 (SD = 0.08), balanced accuracy = 0.55 (SD= 0.07)). No significant differences were found between prediction performances of the 1^st^- or 2^nd^-level classifiers and the dummy classifier using the corrected resampled t-test.

Re-performing the analyses using CAT12-extracted regional thickness and gyrification as structural data resulted in a 1^st^-level AUROC = 0.62 (SD = 0.08), balanced accuracy = 0.56 (SD = 0.06) (corrected resampled t-test p = 0.56). The 2^nd^-level voting classifier using prediction probabilities of all 1^st^-level classifiers as input features resulted in an AUROC = 0.64, (SD = 0.08), balanced accuracy = 0.56 (SD = 0.06) (p = 0.48). The 2^nd^-level Random Forest classifier resulting in an AUROC = 0.60 (SD = 0.08), balanced accuracy = 0.56 (SD = 0.06) (p= 0.71).

**Supplementary Table 1: Pre-treatment patient sample description of follow-up responders and nonresponders**

|  | Follow-up responders | Follow-up non-responders | p-value |
| --- | --- | --- | --- |
| Variables | N=143 | N=40 |  |
| *Demographic characteristics at pre-treatment* | | | |
| Gender (m/f) | 19/124 | 5/35 | n.s. |
| Site distribution | WÜ: 67  MS: 76 | WÜ: 16  MS: 23 | n.s. |
| Age (SD) | 28.6 (9.2) | 27.9 (8.1) | n.s. |
| Years of education (SD) | 14.7 (3.0) | 14.7 (3.0) | n.s. |
| *Clinical characteristics at pre-treatment* | | | |
| Age of onset spider phobia (SD) | 7.2 (4.7) | 6.2 (4.4) | n.s. |
| Comorbid major depression n% |  |  | n.s. |
| SPQ (SD) | 20.3 (3.9) | 20.9 (3.8) | n.s. |
| LSAS (SD) | 23.8 (17.3) | 26.3 (18.3) | n.s. |
| ASI-3 (SD) | 15.0 (7.4) | 16.3 (8.4) | n.s. |
| STAI trait (SD) | 34.6 (8.3) | 37.9 (7.9) | n.s. |
| BDI-II total (SD) | 2.9 (3.4) | 4.3 (4.5) | n.s. |
| UI-18 (SD) | 37.5 (12.4) | 42.5 (14.1) | 4.6e-2* |
| Promis specific phobia (SD) | 11.1 (8.8) | 10.7 (7.6) | n.s. |
| FEAS anxiety (SD) | 101.9 (11.7) | 100.7 (14.8) | n.s. |
| FAS (SD) | 83.2 (12.6) | 85.2 (10.7) | n.s. |
| Final BAT distance (cm) (SD) | 169.1 (67.2) | 165.5 (60.6) | n.s. |
| *Post-treatment* | | | |
| SPQ (SD) | 14.5 (3.1) | 17.8 (1.9) | 1.8e-12* |
| *Follow-up* | | | |
| SPQ (SD) | 12.4 (2.6) | 17.7 (1.6) | < 2.2e-16* |

Continuous variables with two-sided t-test. Categorical variables with chi-squared test.

WÜ: Würzburg; MS: Münster; SPQ: Spider Fear Questionnaire; LSAS: Liebowitz Social Anxiety Scale; ASI-3; Anxiety Sensitivity Scale 3; STAI: State-Trait Anxiety Inventory; BDI-II: Beck Depression Inventory II; UI-18: Unsicherheitsintoleranz (intolerance of uncertainty) 18 scale; PROMIS = Patient-Reported Outcomes Measurement Information System (PROMISPHO: specific phobia); FEAS: Fragebogen zur Ekel und Angst vor Spinnen (questionnaire regarding disgust and fear of spiders); FAS: Fragebogen zur Angst von Spinnen (questionnaire regarding the fear of spiders); BAT: Behavioural avoidance test.


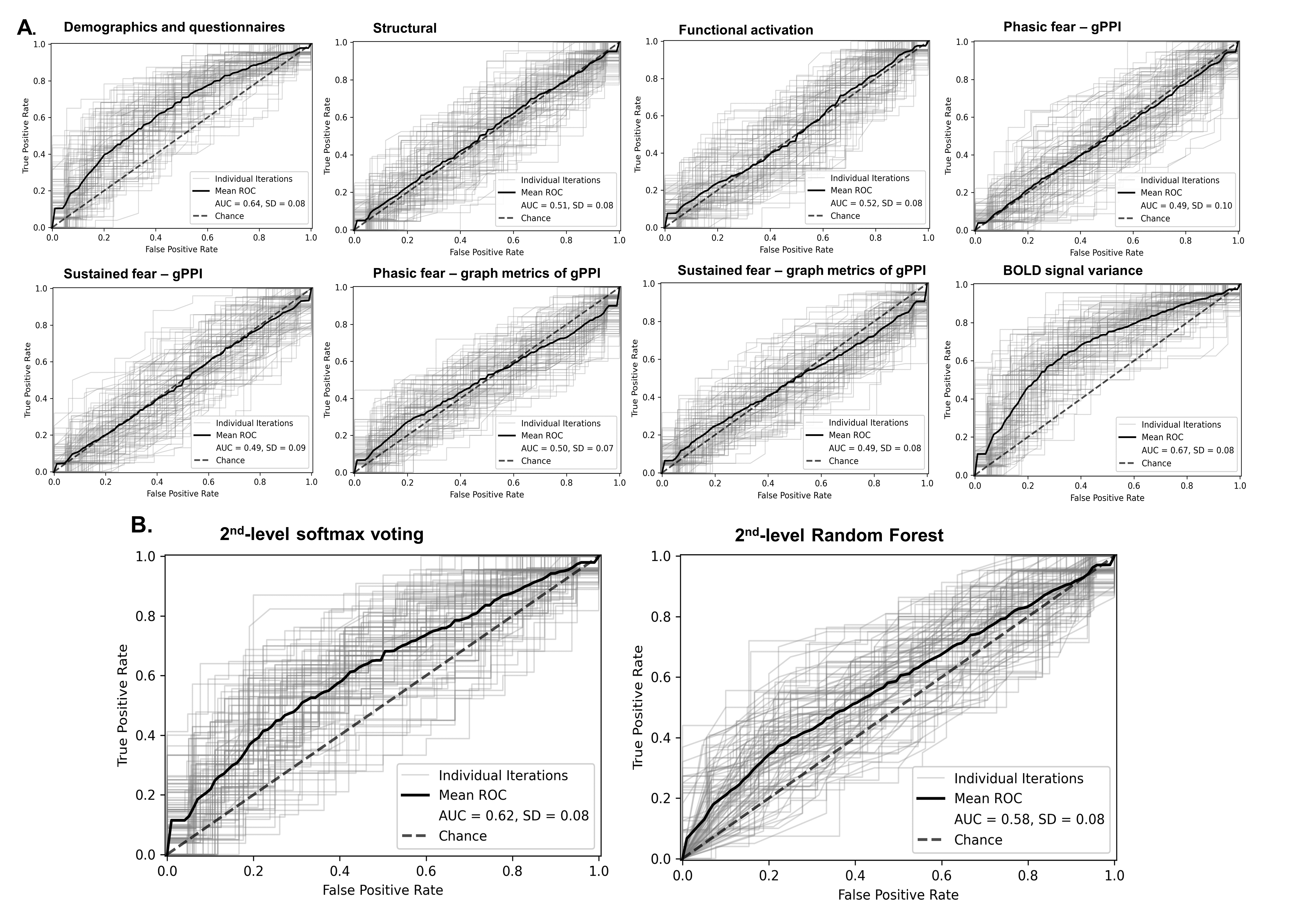
**Supplementary Figure 1: Area under the receiving operating curves for treatment outcome classification using functional features extracted for regions of interest across the whole brain.**

A: 1^st^-level classification results; B: 2^nd^-level classification results.

gPPI: generalized psychophysiological interactions.

**Supplementary References**

1. Klorman R, Weerts TC, Hastings JE, Melamed BG, Lang PJ. Psychometric description of some specific-fear questionnaires. Behav Ther. 1974 May 1;5(3):401–9.

2. Kemper CJ, Ziegler M, Taylor S. Überprüfung der psychometrischen Qualität der deutschen Version des Angstsensitivitätsindex-3. Diagnostica. 2009 Oct;55(4):223–33.

3. Heimberg RG, Horner KJ, Juster HR, Safren SA, Brown EJ, Schneier FR, et al. Psychometric properties of the Liebowitz Social Anxiety Scale. Psychol Med. 1999 Jan;29(1):199–212.

4. Gerlach AL, Andor T, Patzelt J. Die Bedeutung von Unsicherheitsintoleranz für die Generalisierte Angststörung Modellüberlegungen und Entwicklung einer deutschen Version der Unsicherheitsintoleranz-Skala. Z Für Klin Psychol Psychother. 2008 Jul;37(3):190–9.

5. Laux L. Das State-Trait-Angstinventar (STAI): theoretische grundlagen und handanweisung.

6. Rinck M, Bundschuh S, Engler S, Muller A, Wissmann J, Ellwart T, Becker ES. Reliability and validity of German versions of three instruments measuring fear of spiders. Diagn Informationsorgan Über Psychol Tests Untersuchungsmethoden. 2002;48:141–9.

7. Schaller E., Gerdes A., Alpers G. W. Angst ungleich Ekel: Der Fragebogen zu Ekel und Angst vor Spinnen. (Wissenschaftliche Beiträge zum 24. Symposium der Fachgruppe Klinische Psychologie und Psychotherapie (Vol. 105), Lengerich).

8. Krohne HW, Egloff B, Kohlmann CW, Tausch A. Untersuchungen mit einer deutschen version der" positive and negative affect schedule"(PANAS). Diagn-Gottingen-. 1996 Jan 1;42:139–56.

9. Stöber J. Reliability and validity of two widely-used worry questionnaires: self-report and self-peer convergence. Personal Individ Differ. 1998 Jun 1;24(6):887–90.

10. Fydrich T. SPAI-Soziale Phobie und Angst Inventar. Diagn Verfahr Psychother Gött Hogrefe. (2002):335–8.

11. Ehlers A, Margraf J, Chambless. Fragebogen zu körperbezogenen Ängsten, Kognitionen und Vermeidung: AKV. Beltz-Test. 2001;

12. Hautzinger M, Keller F, Kühner C. Das Beck Depressionsinventar II. Dtsch Bearb Handb Zum BDI II. 2006;

13. Radloff LS. The CES-D Scale: A Self-Report Depression Scale for Research in the General Population. Appl Psychol Meas. 1977 Jun 1;1(3):385–401.

14. Flor H. Kurzer Fragebogen zur Erfassung von Belastungen (KFB) In Huber (Ed.). Psychobiol Schmerzes Huber Bern.

15. Cella D, Riley W, Stone A, Rothrock N, Reeve B, Yount S, et al. The Patient-Reported Outcomes Measurement Information System (PROMIS) developed and tested its first wave of adult self-reported health outcome item banks: 2005–2008. J Clin Epidemiol. 2010 Nov 1;63(11):1179–94.

16. Beck AT, Epstein N, Brown G, Steer RA. An inventory for measuring clinical anxiety: psychometric properties. J Consult Clin Psychol. 1988 Dec;56(6):893–7.

17. Carver CS. You want to measure coping but your protocol’s too long: consider the brief COPE. Int J Behav Med. 1997;4(1):92–100.

18. Bernstein DP, Fink L, Handelsman L, Foote J, Lovejoy M, Wenzel K, et al. Initial reliability and validity of a new retrospective measure of child abuse and neglect. Am J Psychiatry. 1994 Aug;151(8):1132–6.

19. Schwarzer R. & Jerusalem M. In: J Weinman, S Wright, & M Johnston, Measures in health psychology: A user’s portfolio. Causal and control beliefs. Windsor, UK: NFER-NELSON; 1995. p. 35–7.

20. Fan L, Li H, Zhuo J, Zhang Y, Wang J, Chen L, et al. The Human Brainnetome Atlas: A New Brain Atlas Based on Connectional Architecture. Cereb Cortex N Y N 1991. 2016 Aug;26(8):3508–26.

21. Keuken MC, Bazin PL, Backhouse K, Beekhuizen S, Himmer L, Kandola A, et al. Effects of aging on T₁, T₂*, and QSM MRI values in the subcortex. Brain Struct Funct. 2017 Aug;222(6):2487–505.

22. Neudorfer C, Germann J, Elias GJB, Gramer R, Boutet A, Lozano AM. A high-resolution in vivo magnetic resonance imaging atlas of the human hypothalamic region. Sci Data. 2020 Sep 15;7(1):305.
